# Combined Anterior and Posterior Decompression With Fusion for Cervical Ossification of the Posterior Longitudinal Ligament

**DOI:** 10.3389/fsurg.2021.730133

**Published:** 2022-01-14

**Authors:** Chao-Hung Kuo, Yi-Hsuan Kuo, Chih-Chang Chang, Hsuan-Kan Chang, Li-Yu Fay, Jau-Ching Wu, Wen-Cheng Huang, Henrich Cheng, Tsung-Hsi Tu

**Affiliations:** ^1^Department of Neurosurgery, Neurological Institute, Taipei Veterans General Hospital, Taipei, Taiwan; ^2^School of Medicine, National Yang Ming Chiao Tung University, Taipei, Taiwan; ^3^Department of Biomedical Engineering, School of Biomedical Science and Engineering, National Yang Ming Chiao Tung University, Taipei, Taiwan; ^4^Department of Biomedical Imaging and Radiological Sciences, National Yang Ming Chiao Tung University, Taipei, Taiwan; ^5^Institute of Pharmacology, National Yang Ming Chiao Tung University, Taipei, Taiwan

**Keywords:** anterior decompression and fusion, ACDF, ACCF, circumferential decompression, laminectomy, OPLL

## Abstract

**Objective:**

Cervical myelopathy caused by ossification of the posterior longitudinal ligament (OPLL) is not uncommon among Asian ethnic groups. Despite reports comparing the pros and cons of anterior- and posterior-only approaches, the optimal management remains debatable. This study aimed to evaluate the outcomes of patients who underwent a combined anterior and posterior approach, simultaneous circumferential decompression and fixation, for cervical OPLL.

**Method:**

The study retrospectively reviewed patients with OPLL and who underwent circumferential decompression and fixation, combined anterior corpectomy and posterior laminectomy. The clinical outcomes were evaluated by the Visual Analog Scale of neck and arm pain, the Japanese Orthopedic Association scores, Neck Disability Index, and Nurick scores at each time-point of evaluation. Radiological evaluations included plain and dynamic radiographs and computed tomography for every patient within 2 years post-operation. Subgroup analyses were further performed between the groups, with and without intra-operative cerebrospinal fluid (CSF) leakage.

**Results:**

A total of 41 patients completed the follow-up for more than 2 years (mean = 39.8 months) and were analyzed. Continuous-type OPLL was the most common (44%), followed by segmental (27%), mixed (22%), and localized types (7%) in the cohort. Myelopathy and all other functional outcomes improved significantly at 2 years post-operation (all *p* < 0.05). There were 13 (32%) patients who had intra-operative CSF leakage. At 2 years post-operation, there were no differences in the demographics, functional outcomes, and complication rates between the CSF-leakage and no-leakage groups. The CSF-leakage group had more patients with continuous-type OPLL than the no-leakage group (77 vs. 29%, *p* = 0.004 < 0.05). During the follow-up, there was no secondary or revision surgery for pseudomeningocele, pseudarthrosis, or other surgery-related complications.

**Conclusions:**

Simultaneous circumferential decompression and fixation combine the surgical benefits of sufficient decompression by the posterior approach and direct decompression of OPLL by the anterior approach. It is an effective surgical option for patients with cervical myelopathy caused by OPLL, given that myelopathy unanimously improved without neurological complications in this study. The fusion rates were high, and reoperation rates were low. Despite higher rates of CSF leakage, there were no related long-term sequelae, and minimal wound complications.

## Introduction

Ossification of the posterior longitudinal ligament (OPLL) is a remarkable risk factor that leads to cervical myelopathy, and commonly occurs in countries of East Asia, including Taiwan, Japan, and Korea (1, 2). The pathogenesis of OPLL is still uncertain, and may be associated with genetic and environmental factors (3). According to radiological findings, OPLL is classified as continuous, segmental, localized or mixed types (4). The selection of a surgical strategy, including the anterior-only, the posterior-only, or combined anterior-and-posterior approaches, depends on the imaging findings and clinical conditions.

The anterior-only approach, including cervical discectomy with or without corpectomy and fusion, provides direct decompression for spinal cord compression caused by OPLL. In a meta-analysis study, including 491 patients in six studies, the anterior approach had better post-operative surgical outcomes evaluated by the JOA scores (5). However, the complications of anterior approaches such as dysphagia, cerebrospinal fluid (CSF) leakage, pseudomeningocele, excessive epidural hemorrhage, and spinal cord injury are challenging to manage. On the other hand, the posterior-only approach, such as laminoplasty, or laminectomy with or without instrument fixation/fusion, is an alternative surgical option for patients with cervical spinal stenosis caused by OPLL, which had a pre-operative canal occupying ratio <60% (6). For the patients with a pre-operative canal occupying ratio > 60%, the anterior-only approach had better neurofunctional recovery in JOA scores than the posterior-only approach. However, the anterior-only approach group had a higher rate of complications and more re-operations (5, 6). For the patients with severe myelopathy caused by OPLL, the anterior-only approach had higher surgical risks and complications, which may lead to post-operative neurological deficits and poor surgical outcomes. The posterior-only approach, including decompressive laminectomy with and without internal fixation, provided a surgical option for indirect cord decompression. However, the posterior approach is not suitable for patients with kyphotic cervical alignment and it carries a higher risk of C5 palsy. To safely and effectively decompress the severe spinal stenosis caused by prominent OPLL, the combined anterior and posterior approach first secures the adequate decompression *via* the posterior approach to significantly lower the risk of severe neurological complication in the following anterior approach. The combined approach also provides 360 degrees fixation for the long segment anterior corpectomy in the multi-level OPLL and also provides a better fusion rate and a lower instrumentation failure rate in the osteoporotic condition, which is not rare in patients with OPLL.

For the continuous type or multi-level type of OPLL, circumferential decompression and fixation would be a reasonable surgical strategy, which includes posterior laminectomy with instrument fixation and fusion, and anterior cervical corpectomy/discectomy and fusion. This maneuver combines advantages of the anterior-only and posterior-only approaches. The dura sac can be well-decompressed from the posterior laminectomy to create space that makes the anterior approach safer with less complications (7, 8). In this study, we analyzed this combined approach for patients with cervical myelopathy caused by OPLL, and evaluated clinical and functional outcomes perioperatively and at 2-years follow-up.

## Methods

### Patient Population

The patients who underwent surgery for cervical ossification of the posterior longitudinal ligament (OPLL) at a single medical center were reviewed retrospectively. OPLL was diagnosed and confirmed by computed topography (CT) pre-operatively. All patients had received at least 12 weeks of conservative therapy, including medications and physical therapy, prior to the surgery. Surgical indication in this study was medically refractory cervical radiculopathy with or without myelopathy that was caused by OPLL. All patients underwent circumferential surgery that included posterior laminectomy with fixation, and anterior decompression (discectomy and corpectomy) with fusion, respectively. The patients with any one of the following conditions would be not considered: traumatic ligamentous or bony injury, infection, or malignancy. Furthermore, patients who had prior anterior cervical spine surgery or those with less than 2-years of or loss to follow-up were also excluded. All pre- and post-operative clinical and radiological evaluations were analyzed.

This study was approved by our institutional ethics committee and patient consent was obtained. Written informed consent was obtained from the individuals for the publication of any potentially identifiable images or data included in this article.

### Surgical Technique

Surgical interventions were typically preceded by the posterior approach of laminectomy and fixation, and followed by the anterior approach for decompression of OPLL. Surgical levels were decided by the levels of cord compression caused by OPLL, based on radiological examinations, and neurological presentations from physical examinations.

All patients were placed prone under general anesthesia with the neck maintained and fixed by a Mayfield clamp in a neutral to slightly lordotic position. After sterilization and adequate cushion, a linear incision was made following the midline of the posterior neck. Cervical stenosis that caused OPLL was decompressed posteriorly by laminectomy, and instrumented by lateral mass screws (for C1, or C3-C7 levels), pedicle screws (for C2 or C7 levels), and pars screws (for C2 level) under C-arm guidance. Intraoperative fluoroscopy was applied for close checking of the position of instrumentation. After checking bleeders, the wound was closed layer by layer. The patient was repositioned supine for the anterior approach.

A horizontal skin incision was made following one of the skin creases in the right neck according to the operated level. The standard anterior cervical approach was adapted for exposure of the target level with self-retaining retractors. The neural elements were well decompressed under the microscope by removal of the intervertebral disc and vertebral bodies with OPLL. Proper sizing, accurate midline acquisition, and adequate endplate coverage were the major principles for restoration of the cervical alignment. The implant was confirmed intraoperatively by lateral fluoroscopy for the optimal position. After copious irrigation and hemostasis, all wounds were subsequently closed in layers with a drainage catheter routinely placed to prevent hematoma formation.

### Clinical and Radiographic Evaluations

Clinical outcomes, including the Visual Analog Scale (VAS) for neck and arm pain, clinical symptom scores of the Japanese Orthopedic Association (JOA), Neck Disability Index (NDI), and Nurick scores were reviewed retrospectively, and measured by a questionnaire completed pre-operatively and at ~6, 12, 18 and 24 months post-operatively. Radiological evaluations, including radiographs, CT, and MRI scans were performed pre-operatively and at the specified time points of post-operative follow-up. These included Type of OPLL, including continuous, segmental, mixed and localized, and were classified pre-operatively and confirmed by cervical CT scans. All quantitative measurements were analyzed by the commercial software, SmartIris (Taiwan Electronic Data Processing Co., Taiwan), and interpreted independently by radiologists and neurosurgeons. If any ambiguity occurred, determination would be made based on a consensus after group discussion.

Peri-operative complications, including neurological deficits, C5 palsy, hoarseness, dysphagia, wound infection, and CSF leakage, were reviewed according to medical records. Subgroup analysis, based on the presence of intra-operative CSF leakage, was performed to analyze its effects on the clinical outcomes.

### Statistical Analysis

All statistical tests were two-tailed. Statistical significance was defined by a *p* < 0.05 at (un)paired *t*-test for continuous variables, or chi-square test for categorical variables, and analyzed by MATLAB (Mathworks, Natick, MA, United States).

## Results

### Demographics

A total of 41 patients who underwent circumferential decompression and fixation for cervical OPLL were included in this study. There was a mean follow-up of 39.8 months; the average age of operated patients was 59.7 years old; and the cohort was male predominant (M:F = 26:15).The case numbers and percentages of continuous, segmental, mixed, and localized types of OPLL were 18 (44%), 11 (27%), 9 (22%), and 3 (7%), respectively (Table 1).

**Table 1:** Demography of the OPLL patients.

**Characteristics**	**Value**
**No. of patients**	41
**Age (years)^†^**	59.7 ± 10.3
**Sex (F:M)**	15: 26
**Diabetes mellitus**	8
**Hypertension**	12
**Smoking**	2
**Levels of surgery**	
3 levels	1
4 levels	8
5 levels	18
6 levels	5
7 levels	9
**Type of OPLL**	
Continuous	18
Segmental	11
Mixed	9
Localized	3
**Mean follow-up (months)^†^**	39.8 ± 20.3

† *Values are presented as mean ± SD*.

### Peri-Operative Clinical Outcomes

**Figure 1:**
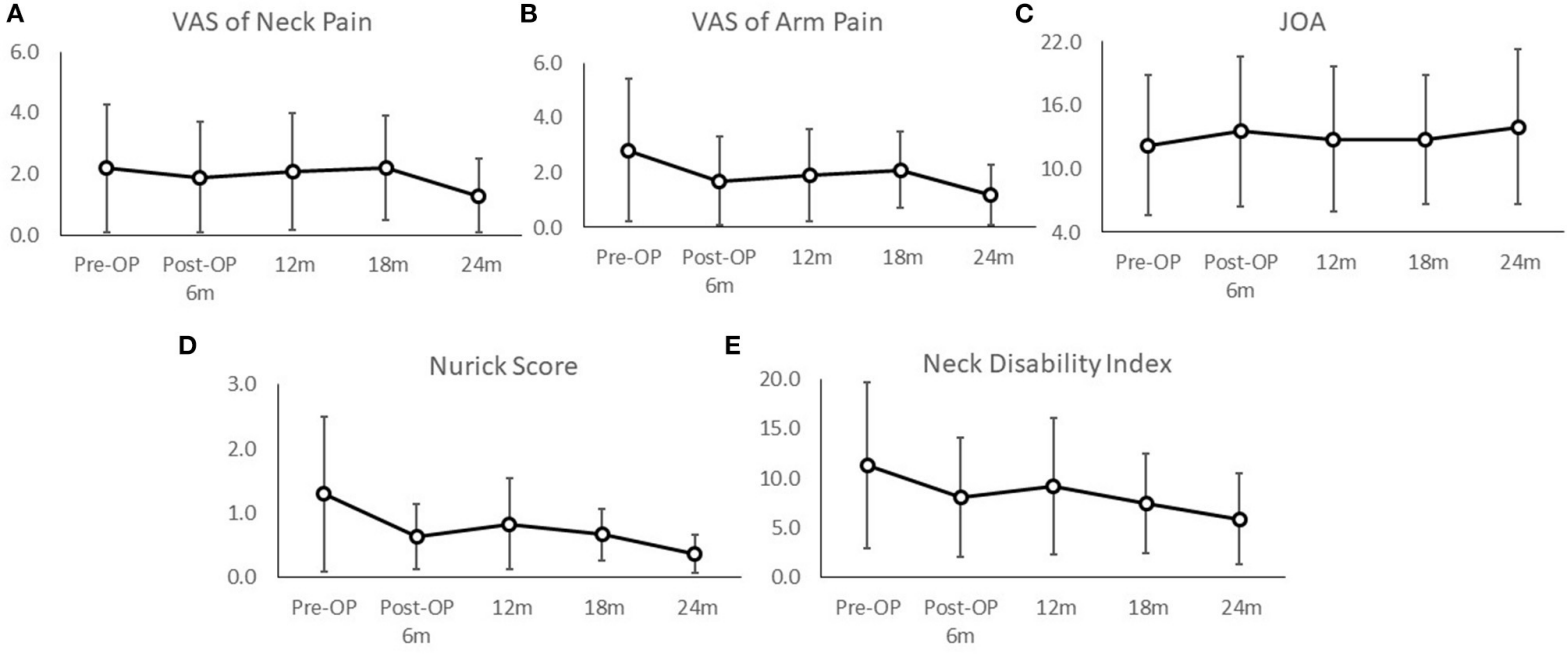
The peri-operative and post-operative functional outcomes included the VAS scores of neck **(A)** and arm pain **(B)**, JOA scores **(C)**, Nurick scores **(D)**, and neck disability index **(E)**. All post-operative outcomes at 2-years follow-up had significant improvement (*p* < 0.05), compared with pre-operative status. The value of each follow-up time point was illustrated by mean +/–standard error.

The patient-reported outcomes were analyzed during the peri-operative periods. The VAS score for arm pain improved significantly from 2.8 ± 2.6 to 1.2 ± 1.4 (pre- and post-operative, respectively, *p* = 0.04 < 0.05, Figure 1A), and the VAS score for neck pain also improved from 2.2 ± 2.2 to 1.3 ± 1.44 (pre- and post-operative, respectively, *p* = 0.28, Figure 1B). The JOA scores at post-operative 2-year follow-up had significant improvement, compared with the pre-operative ones (12.2 ± 6.6 to 14.0 ± 7.3, *p* = 0.01 < 0.05, Figure 1C), as well as Nurick scores (1.3 ± 1.2 to 0.4 ± 0.4, *p* = 0.01 < 0.05, Figure 1D), and NDI (11.3 ± 8.3 to 5.9 ± 4.6, *p* = 0.001 < 0.05, Figure 1E, Table 2).

**Table 2:** Pre- and post-operative clinical characteristics.

**Characteristics**	**Value**
**Pre-operative functional evaluations^†^**	
VAS (neck pain)	2.4 ± 2.0
VAS (arm pain)	2.8 ± 2.6
JOA	12.2 ± 6.6
NDI	11.3 ± 8.3
Nurick score	1.3 ± 1.2
**Post-operative functional evaluations^†^**	
VAS (neck pain)	1.3 ± 1.4
VAS (arm pain)	1.2 ± 1.4
JOA	14.0 ± 7.3
NDI	5.9 ± 4.6
Nurick score	0.4 ± 0.4
**Peri-operative complications**	
Neurological deficit	0
Transient C5 palsy	12
Unilateral	11
Bilateral	1
Transient hoarseness	4
Transient dysphagia	0
Wound infection	3
CSF leakage	13
Secondary surgery (%)	0

† *Values are presented as mean ± SD*.

Twenty-five patients (25/41, 61%) had at least one of the following complications after surgery: neurological deficit, C5 palsy, transient hoarseness, transient dysphagia, wound infection, and CSF leakage. No patient had permanent neurological deficit. There were 12 patients with unilateral and one patient with bilateral C5 palsies, all of which were transient, and the patients recovered within 6 months. There were four patients with post-operative transient hoarseness, and three patients with wound infection. Intra-operative CSF leakage in the anterior approach occurred in 13 patients (13/41 = 32%). No patient required lumbar CSF drainage intra- or post-operation. No patient needed secondary surgery due to complication during the follow-up period (Table 2).

**Figure 2:**
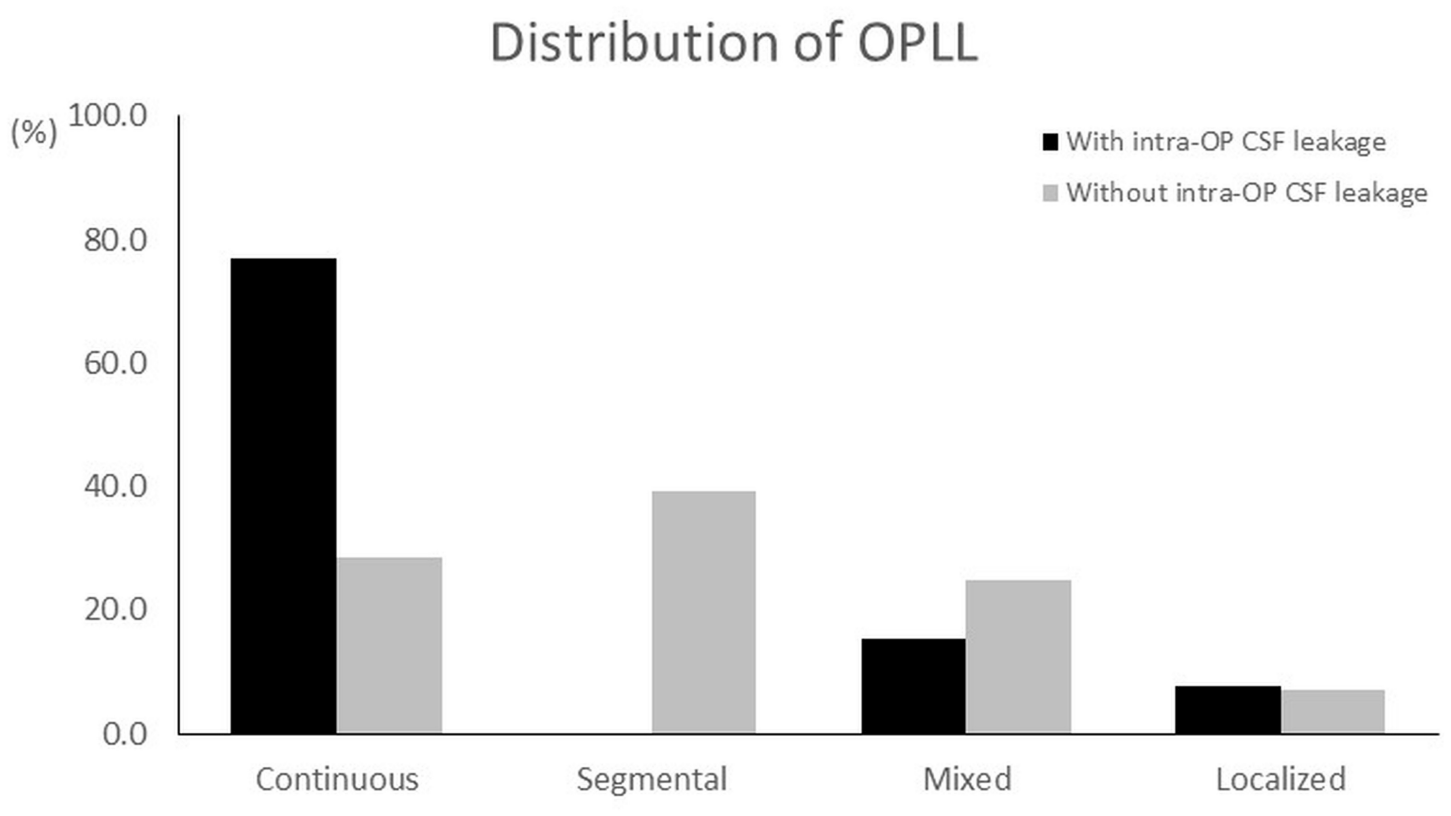
Comparison of type of OPLL between the groups with and without intra-operative CSF leakage. In the group with intra-operative CSF leakage, the continuous type of OPLL had the highest percentage (77%), followed by the mixed type (15%), localized (8%), and segmental (0%). For the group without intra-operative CSF leakage, the segmental type of OPLL had a higher percentage (39%) than the others; the percentages of the continuous, mixed, and localized types were: 29, 25, and 7%, respectively.

Subgroup analysis was performed to compare the demographic data and clinical outcomes of patients with or without intra-operative CSF leakage (13 vs. 28 patients, respectively, Table 3). There was no difference between the two groups in age, sex distribution, or time of follow-up (all *p* > 0.05). For the group with intra-operative CSF leakage, the continuous type of OPLL was predominant; the percentages of the continuous, segmental, mixed, and localized types were: 77, 0, 15, and 8%, respectively (Figure 2). The segmental type OPLL was the majority in the group without intra-operative CSF leakage; the percentage of the continuous, segmental, mixed, and localized types were: 29, 39, 25, and 7%, respectively (Figure 2). Regarding post-operative complications, the group with intra-operative CSF leakage had a higher percentage of transient hoarseness and C5 palsy than those in the group without CSF leakage (38.5 vs. 25%, and 15.4% vs. 7.1% for transient C5 palsy and hoarseness, respectively), but the differences were not statistically significant (all *p* > 0.05, Table 3).

**Table 3:** Comparison of characteristics between the patients with and without intra-operative CSF leakage.

**Characteristics**	**Intra-operative CSF leakage**		***P*-value**
	**Yes**	**No**	
**No. of patients**	13	28	
**Age (years)**	56.9 ± 10.7	61.0 ± 10.1	0.26
**Sex (F:M)**	9:4	17:11	0.86
**Mean of follow-up (month)^†^**	40.9 ± 22.1	39.3 ± 19.8	0.82
**Post-operative functional evaluations^†^**			
VAS (neck pain)	0.7 ± 1.5	1.7 ± 1.9	0.24
VAS (arm pain)	0.7 ± 1.5	1.7 ± 2.1	0.37
JOA	14.7 ± 1.8	13.6 ± 2.7	0.26
NDI	4.0 ± 4.3	6.8 ± 5.4	0.26
Nurick score	0.1 ± 0.4	0.5 ± 0.5	0.1
**Peri-operative complication (%)**			
Transient C5 palsy	38.4 (5/13)	25 (7/28)	0.61
Transient hoarseness	15.4 (2/13)	7.1 (2/28)	0.79
Wound infection	0 (0/13)	10.7 (3/28)	0.57

† *Values are presented as mean ± SD*.

### Case Illustration

**Figure 3:**
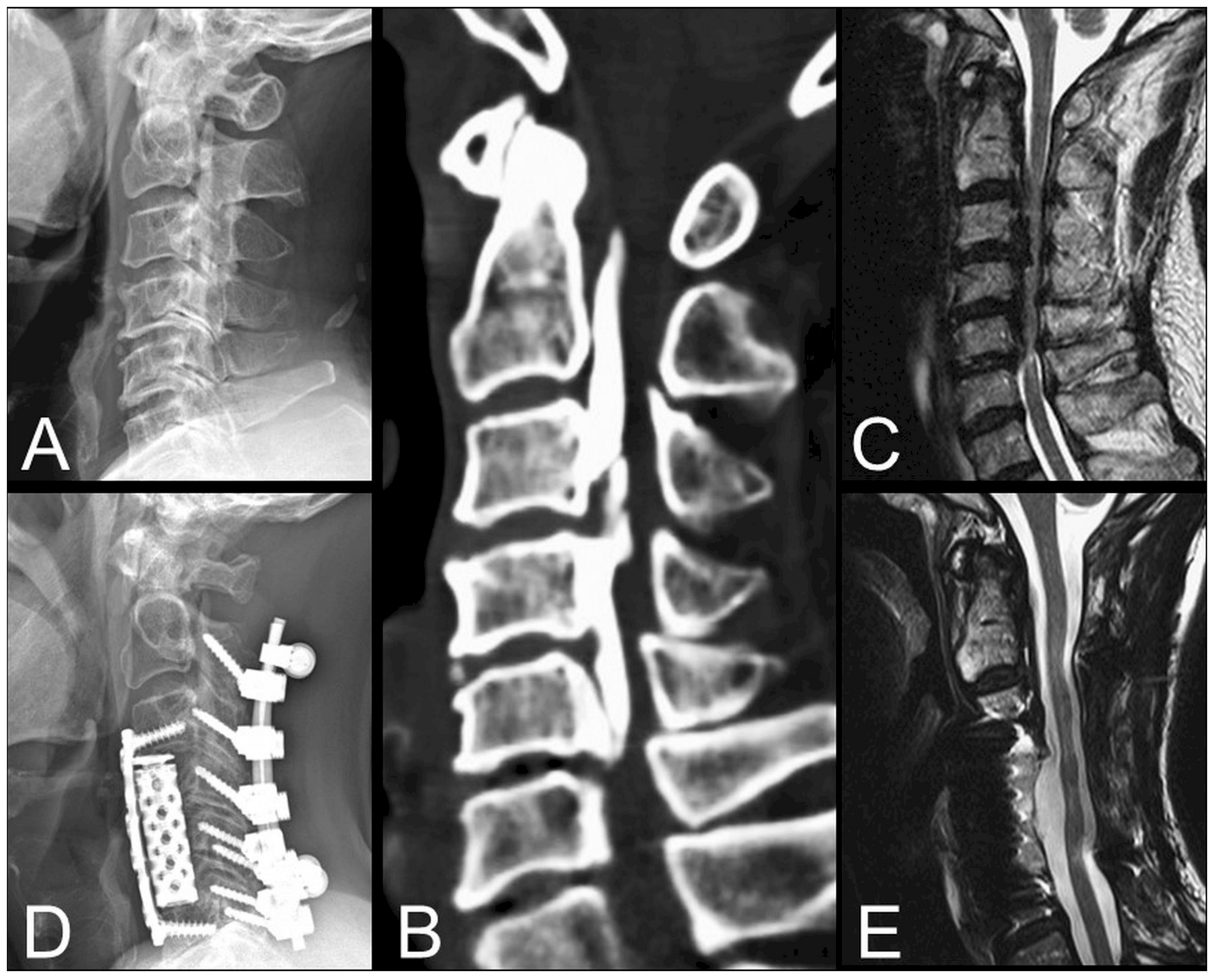
Illustration of a 61-year-old male who presented with progressive bilateral clumsiness of the hands, lower limb weakness, and unsteady gait for months. The lateral view of the cervical radiograph **(A)** and cervical CT scan **(B)** revealed the continuous type OPLL extending from C2 down to C5. The sagittal view of the cervical MRI also revealed severe compressive myelopathy from C2-6 **(C)**. Surgical intervention by circumferential decompression and fixation, including C2-C6 total laminectomy with posterior fixation, and C4-5 corpectomy with fusion, was performed **(D)**. The MRI at 2-years follow-up demonstrated good decompression of the spinal cord **(E)**.

A 61-year-old male presented with progressive clumsiness of the bilateral hands, lower limb weakness, and unsteady gait for months. The lateral view of the cervical radiograph (Figure 3A) and cervical CT scan (Figure 3B) revealed continuous type OPLL extending from C2 to C5. The sagittal view of the cervical MRI revealed severe compressive myelopathy from C2-6 (Figure 3C). Surgical intervention of circumferential decompression and fixation, including C2-C6 total laminectomy with posterior fixation, and C4-5 corpectomy with fusion, was performed (Figure 3D). After surgery, the patient's hand dexterity and lower limb muscle power improved significantly, and MRI revealed good decompression of the spinal cord at 2-years follow-up (Figure 3E).

## Discussion

In this study we investigated the clinical outcomes of 41 cervical OPLL patients treated by circumferential decompression and fixation for cervical myelopathy. All patients were followed up for at least 2 years. The continuous type (44%) of OPLL was the most common in this cohort, followed by the segmental (27%), mixed (22%), and localized (7%) types. Post-operative functional outcomes, including VAS arm pain scores, the JOA scores, and Nurick scores, had significant improvement compared to pre-operative status. Thirteen of all patients had intra-operative CSF leakage due to dura tear. Subgroup analysis was performed to compare the patients with (13/41) and without (28/41) intraoperative CSF leakage. The demographic data were not different between the two groups, but the continuous type of OPLL predominated in the group that had intraoperative CSF leakage. Complications including postoperative transient neurological deficit or hoarseness were not correlated with intraoperative CSF leakage. During the period of follow-up, there was no patient who received secondary surgery.

The selection of approaches for cervical OPLL remained debatable. For the anterior-only approach, it provided direct decompression of the spinal cord by removing OPLL, reduced progression of OPLL, better maintained the lordotic curve, and had less extensive tissue dissection. However, the anterior-only approach was technically demanding, and had a higher rate of CSF leakage due to dura tear (9). The posterior-only approach, especially laminoplasty, preserved more ROM than the anterior-only approach, but provided indirect decompression of the spinal cord. There were higher rates of C5 palsy in the posterior-only approach (10). In a study that included 913 patients with OPLL, 71 and 64 patients underwent the anterior-only and the posterior-only (laminoplasty) approach, respectively (11). The study concluded that the anterior-only approach was suitable for patients with an occupying ratio > 60% or with increased intramedullary signal intensity on MRI images (11, 12). Post-operative recovery was not related to anterior- or posterior-only approaches, but associated with older age, the grade of intramedullary signal change, and occupying ratio (11). In a randomized control study including 56 OPLL patients, 27 patients received anterior decompression and fusion, and 29 patients underwent posterior laminectomy. The report concluded that the anterior approach provided more improvement than the posterior approach among OPLL patients regardless of intramedullary signal intensity change on MRI (13). Although the anterior-only approach could remove OPLL and decompress the compressed spinal cord directly, intra-operative dura tears may cause cord damage and severe neurological deficit, and the hemorrhage may be profound.

The posterior-only approach for OPLL included laminoplasty and laminectomy with fusion. The superiority of laminoplasty or laminectomy remains controversial (10), but the consistent finds across different studies indicate a higher incidence rate of C5 palsy in laminectomy. A meta-analysis study included 429 patients who underwent laminoplasty and 101 patients who underwent laminectomy with fusion to investigate the progression of OPLL after the posterior-only approach. The overall rate of OPLL progression increased with time (60% at 10-years follow-up), and a higher rate of OPLL progression was noted in the group of laminoplasty (62.5%), compared with the fusion group (7.6%) (14). Laminoplasty for treating patients with OPLL may be less favorable in view of OPLL progression and clinical deterioration by time. Considering spinal instability as a significant factor in the pathogenesis of OPLL, a study reported 52 patients with OPLL who were treated by spinal fixation without any form of bone or soft tissue decompression. The surgical results revealed successful arthrodesis in the surgically treated segments with clinical improvement (15). More research is needed to evaluate the clinical outcomes of the posterior-only fixation with and without decompression in patients with severe myelopathy caused by OPLL.

For the patients with OPLL progression or kyphotic change of cervical alignment after the posterior-only approach, anterior decompression and fusion provided an option for revision surgery for those with neurological deterioration. In a study including 19 patients who underwent revision anterior surgery, and for a mean of 2-years follow-up, the results revealed a low clinical improvement rate, and a high incidence of intra-operative CSF leakage (8/19 = 42%). Neurological function worsened in five patients (26%) (16). These findings implied the advantages of circumferential decompression and fixation for the patients with OPLL. The circumferential surgical strategy could combine the benefits of generous decompression of the spinal cord and mitigation of OPLL progression by posterior fixation and direct decompression of OPLL by the anterior approach. The posterior-approach was the first step to decompress the spinal cord. A decompressed spinal cord tends to slightly expand dorsally and thus significantly decrease the surgical risk, such as cord injury, while removal of OPLL is possible from the anterior approach. Thus, the complication rates of this combined approach, such as C5 palsy or intra-operative CSF, were not higher than other published studies.

There were several limitations to this study. The retrospective study only provided single arm comparison between pre- and post-operative outcomes. Studies enrolling more patients with longer follow-up would be needed to further corroborate the results of this study.

## Conclusion

Simultaneous circumferential decompression and fixation combine the benefits of generoussufficient decompression of the spinal cord and mitigation of OPLL progression by posterior fixation and direct decompression of OPLL by the anterior approach. It is an effective surgical option for patients with cervical myelopathy caused by OPLL, given that myelopathy unanimously improved in this study. The merits of this combined approach would be even more eminent in cases of long segment OPLL with severe stenosis and should be considered in the decision making of surgical approaches. Despite high rates of CSF leakage, there were minimal wound complications, the fusion rates were high, and reoperation rates were low.

## Data Availability Statement

The datasets presented in this article are not readily available due to the protection of patients' privacy. Requests to access the datasets should be directed to Chao-Hung Kuo, chaohungk@gmail.com.

## Ethics Statement

The studies involving human participants were reviewed and approved by Taipei Veterans General Hospital. The patients/participants provided their written informed consent to participate in this study. Written informed consent was obtained from the individuals for the publication of any potentially identifiable images or data included in this article.

## Author Contributions

C-HK, J-CW, and T-HT designed the study, analyzed the data, wrote, and reviewed the manuscript. Y-HK, C-CC, H-KC, and L-YF participated in data collection. J-CW, W-CH, and HC provided the data resource. All authors reviewed and approved the manuscript.

## Conflict of Interest

The authors declare that the research was conducted in the absence of any commercial or financial relationships that could be construed as a potential conflict of interest.

## Publisher's Note

All claims expressed in this article are solely those of the authors and do not necessarily represent those of their affiliated organizations, or those of the publisher, the editors and the reviewers. Any product that may be evaluated in this article, or claim that may be made by its manufacturer, is not guaranteed or endorsed by the publisher.
